# Unsuccessful therapy with adefovir and entecavir-tenofovir in a patient with chronic hepatitis B infection with previous resistance to lamivudine: a fourteen-year evolution of hepatitis B virus mutations

**DOI:** 10.1186/1471-2334-11-178

**Published:** 2011-06-22

**Authors:** Lucila Cassino, Silvina Benetti, Fabian Fay, Hugo Tanno, Jorge Quarleri

**Affiliations:** 1 Centro Nacional de Referencia para el SIDA, Departamento de Microbiología, Facultad de Medicina, Universidad de Buenos Aires, Argentina; 2 Consejo Nacional de Investigaciones Científicas y Técnicas, Argentina; 3 Centro de Diagnóstico Médico de Alta Complejidad (CIBIC), Rosario, Argentina; 4 Servicio de Gastroenterología y Hepatología del Hospital Provincial de Centenario, Rosario, Argentina

**Keywords:** hepatitis B virus, lamivudine, resistance, tenofovir, entecavir

## Abstract

**Background:**

Complex mutants can be selected under sequential selective pressure by HBV therapy. To determine hepatitis B virus genomic evolution during antiviral therapy we characterized the HBV quasi-species in a patient who did no respond to therapy following lamivudine breakthrough for a period of 14 years.

**Case Presentation:**

The polymerase and precore/core genes were amplified and sequenced at determined intervals in a period of 14 years. HBV viral load and HBeAg/Anti-HBe serological profiles as well as amino transferase levels were also measured. A mixture of lamivudine-resistant genotype A2 HBV strains harboring the rtM204V mutation coexisted in the patient following viral breakthrough to lamivudine. The L180M+M204V dominant mutant displayed strong lamivudine-resistance. As therapy was changed to adefovir, then to entecavir, and finally to entecavir-tenofovir the viral load showed fluctuations but lamivudine-resistant strains continued to be selected, with minor contributions to the HBV quasi-species composition of additional resistance-associated mutations. At the end of the 14-year follow up period, high viral loads were predominant, with viral strains harboring the lamivudine-resistance signature rtL180M+M204V. The precore/core frame A1762T and G1764A double mutation was detected before treatment and remaining in this condition during the entire follow-up. Specific entecavir and tenofovir primary resistance-associated mutations were not detected at any time. Plasma concentrations of tenofovir indicated adequate metabolism of the drug.

**Conclusions:**

We report the selection of HBV mutants carrying well-defined primary resistance mutations that escaped lamivudine in a fourteen-year follow-up period. With the exception of tenofovir resistance mutations, subsequent unselected primary resistance mutations were detected as minor populations into the HBV quasispecies composition during adefovir or entecavir monotherapies. Although tenofovir is considered an appropriate therapeutic alternative for the treatment of entecavir-unresponsive patients, its use was not effective in the case reported here.

## Background

Hepatitis B infection affects two billion people worldwide and nearly 350 million individuals are chronically infected. If left untreated, about one-third will develop progressive and possibly fatal liver disease [1]. Drugs inhibiting viral replication achieve higher treatment response compared to IFN-[alpha] or pegylated-IFN-[alpha], although relapse is common when treatment is interrupted. Treatment with lamivudine, a viral polymerase inhibitor, results in a rapid 4-5 Log_10 _decline in viral load and it has shown to improve liver histology after one-year treatment [2-4]. A major limitation is the emerging resistance mutations within the viral polymerase gene, resulting in a resistance rate of approximately 20% per year [5]. Adefovir dipivoxil is an alternative which develops slow resistance rates compared to lamivudine, with different patterns [6,7].

Another alternative is entecavir, rapidly metabolized to its active triphosphate metabolite [8]. A 7 Log_10 _and a 5 Log_10 _decline is observed when 0.5 mg dose is used in nucleoside-naïve hepatitis B e antigen (HBeAg)-positive and HBeAg-negative patients, respectively. When a double dose is administered (1 mg daily), it is also effective against lamivudine-resistant strains, yet with lower viral load reduction vs. wild type [9-11]. The resistance rates observed with entecavir are still lower considering that HBV DNA rebounds were exhibited in only 2% of nucleoside-naïve patients treated with entecavir for up to 2-year therapy [12]. Such rate was also minor in those lamivudine-refractory patients during 2 years of entecavir treatment [13].

Another drug, tenofovir disoproxil fumarate -an acyclic nucleotide analog reverse transcriptase inhibitor-, showed potent activity in wild-type and lamivudine-resistant HBV in HIV-HBV coinfected and HBV monoinfected patients [14-17].

In this 14-year follow-up study (1996-2010), we described a therapeutic dilemma where adefovir, entecavir and entecavir-tenofovir non-response is progressively observed in a patient that previously experienced lamivudine resistance.

## Case Presentation

A 47-year-old woman (weight: 62 kg) was diagnosed with chronic active HBeAg-positive hepatitis B in 1996. Viral parameters such as HBeAg, antiHBe IgG and HBV plasma viral load from frozen samples were simultaneously measured by a unique operator to reduce intra and inter-assay variations. HBeAg and anti-HBe were determined by electrochemiluminescence (ELECSYS 2010, Roche Diagnostic). HBV viral load levels were determined by real time PCR (COBAS TaqMan Roche Molecular Systems, dynamic range 30 IU/ml to 110,000,000 IU/ml) [18]. Liver inflammation was measured by serum alanine aminotransferase (ALT) levels.

For sequence analysis, HBV pol and preC-core genes were sequentially sequenced with primers as previously described [19]. The analysis was performed using an ABI3100 instrument (Applied Biosystems), and the sequences introduced in this work as well as those obtained from the GenBank database were aligned with ClustalX v1.83 [20] and edited with Bioedit v7.0.9.0 [21]. The HBV genotype was assessed by phylogenetic inference using the Neighbor-joining algorithm with the Kimura-two-parameter model of molecular evolution in the MEGA v3.1 software [22].

**Figure 1:**
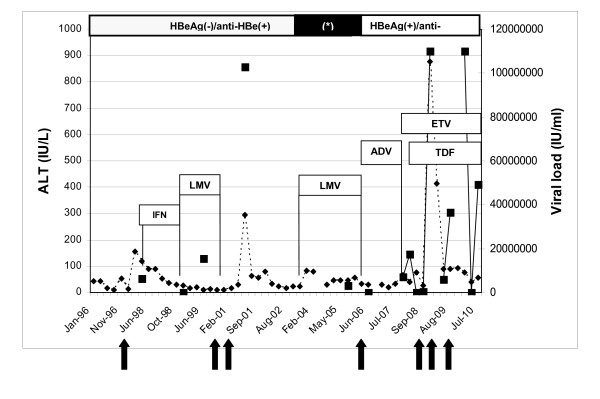
**HBV viral load dynamics (black squares) and course of aminotransferase -ALT- (black triangles) levels during different treatment (IFN: interferon; LMV: lamivudine; ADV: adefovir; ETV: entecavir; TDF: tenofovir)**. In the superior panel the HBeAg/antiHBe profile during the follow-up is depicted; the asterisk (*) denotes the period exhibiting reversion to HBeAg (+) and anti-HBe (-). In the inferior panel, solid black arrows indicate sampling times for HBV pol gene clonal analysis (see also Table 1).

Sequences from both genes were obtained at different time-points during antiviral therapy (Figure 1). A more detailed HBV pol gene clonal analysis was performed on seven selected viral isolates from serum samples harvested during sequential therapies. Each selected PCR product of pol gene was cloned into pGEM-T Easy vector according to the manufacturer's instructions (Promega, Wisconsin, USA) and at least 15 clones were further analyzed by sequencing. This allowed analysing the evolution of viral quasispecies under the successive antiviral pressures based on analysis of the pol gene.

The anti-HBV treatment interventions accompanied by the HBV viral load and ALT level kinetics are depicted in figure 1. Therapy was initiated when HBV viral load rose to 6.5 × 10^6 ^IU/ml. In 1998 when the patient was treated with IFN-[alpha] 5 MU thrice weekly for 30 weeks, the HBV DNA level was lower than the detection limit. This therapy was interrupted and replaced in 1999 by lamivudine (150 mg once a day). After 2 years of continuous treatment, HBV DNA was detectable (15.5 × 10^6 ^IU/ml). HBeAg remained negative with detectable anti-HBe during this period of treatment. In 2001, after a 6-month discontinuation, HBV DNA level evidenced a post-treatment flare (1 × 10^8 ^IU/ml). At the end of 2003 lamivudine therapy was reintroduced (150 mg once daily). It was accompanied by a rare event of reversion to an HBeAg-positive/anti-HBe negative profile, which remained detectable for two years. The viral load reached 3.1 × 10^6 ^IU/ml at this point.

Ten months later, in 2006, the lamivudine regimen was replaced by adefovir monotherapy (10 mg/day). Initially, viral load levels were low (8.5 × 10^4 ^IU/ml) but after 48 weeks of therapy these levels reached 7.15 × 10^6 ^IU/ml. ALT levels were normal during this interval. Reactivation of HBV replication was assumed considering the simultaneous detection of HBeAg/anti-HBe [17] and the high replication detected. The concurrence of HBeAg and anti-HBe does not appear to be uncommon among antiviral treatment-naïve patients, but its prevalence in therapy-experienced patients is unknown. This serological pattern has been related to extensive hepatocyte damage and severe immune-mediated liver injury and consequent dysfunction [23]. Six months later, ALT level slightly increased (1.5× upper-normal limit - UNL -) and entecavir monotherapy was instituted (1 mg daily).

After a 5-month entecavir monotherapy, tenofovir (300 mg once daily) was added to the treatment regimen for dual therapy. The HBV DNA declined to 1 × 10^5 ^IU/ml for four months, but three months later the viral load reached > 1.1 × 10^8 ^IU/ml. Additionally, an increase in the ALT level (20× UNL) denoted liver inflammation. During the following 48 weeks, the viral replication slightly decreased to 6.1 × 10^6 ^IU/ml but rebounded first to 3.6 × 10^7 ^IU/ml and later to > 1.1 × 10^8 ^IU/ml. More recently (second semester 2010), HBV DNA levels fluctuated between 4.3 × 10^4 ^IU/ml and 4.9 × 10^7 ^IU/ml. By the end of the study follow-up, we were able to measure tenofovir concentration in plasma by high performance-liquid chromatography in three consecutive samples 20 hours after administration reaching 71.6 ng/ml ± 47.6 (mean ± SD).

The ALT-AST levels remained slightly elevated (2× UNL) during the last two years of follow-up.

HBV isolates from the patient were ascribed to genotype A2 based on phylogenetic relatedness among partial HBV genomic sequences from both pol and preC-C genes. A further longitudinal genotypic analysis, including quasispecies composition of HBV strains isolated from the patient revealed the presence of resistance mutations based on pol gene sequences (Table 1). At the beginning of lamivudine treatment, HBV was wild-type in all clones. After 2 years under such therapy, this viral population was completely replaced by mutation rtM204I -homogenously exhibiting lamivudine resistance. When this therapy was interrupted for 6 months, quasispecies composition almost exclusively consisted of *wild-type *pol nucleotide sequences, except for a minor contribution (< 10%) showing the adefovir resistance-associated mutation I233V. Once lamivudine therapy was reintroduced, and until entecavir plus tenofovir were administered, the dual lamivudine resistance-associated mutations rtL180M and rtM204I were invariably detected. Under the latter therapeutic scheme, only ephemerally and with minor contribution (~5%), the A181T and T184L lamivudine-adefovir and entecavir resistance-associated mutations were detected, respectively.

**Table 1:** Hepatitis B virus polymerase gene variants characterized by clonal analysis during the follow-up

Sampling date	Treatment	Resistance mutations*
Jun-98	IFN	*wt*

Feb-01	LMV	(15/15) **M204I**

May-01	No	(14/15) *wt *+ (1/15) I233V

May-02	No	ND

Nov-03	LMV	ND

Sep-04	LMV	ND

Ago-05	LMV	ND

Jul-06	ADF	(15/15) **L180M/M204I**

Dec-08	ETV+TDF	(17/18) **L180M/M204I **+ (1/18) T184L

May-09	ETV+TDF	(13/15) **L180M/M204I **+ (1/15) T184L + (1/15) A181T

Nov-09	ETV+TDF	(15/15) **L180M/M204I**

(*) The proportion of clones is shown between parentheses. The resistance associated mutations detected with prevalence > 20% are bolded; *wt*: wild type; ND: not done

Additionally, another two variations were detected in the reverse transcriptase catalytic domain at the time of lamivudine resistance. These rtA200V and rtI253V variants remained present during entecavir therapy, and were also detected in samples sequenced after breakthrough during entecavir therapy.

The HBV genomic characterization at preC-C level showed the presence of A1762T and G1764A double mutation that was early detected when the patient was untreated, remaining in this condition during the entire follow-up. The T1753C mutation was also consistently detected. The presence of these core promoter mutations are also related to the above-mentioned HBeAg-antiHBe concurrent profile found in this patient, as recently reported [23].

## Discussion

In this study, we described the dynamics of HBV genomic changes and other related parameters (ALT level, HBeAg/antiHBe profile and viral load) during a 14-year period that included sequential monotherapy with lamivudine, adefovir, entecavir, and entecavir-tenofovir in a chronically HBV-infected patient. This patient initially treated with interferon, later consecutively resistant to lamivudine, adefovir, and entecavir -either as monotherapy or combined with tenofovir- showed viral and biochemical resistance with higher HBV DNA levels and only transient viral suppression. It was expected to find viral replication impairment when rtA181T mutation was detected under adefovir monotherapy, since this variant has a secretory defect and exerts a dominant negative effect on wild-type HBV virion secretion. Thus, this mutation is usually detected as a mixed population with wild-type [24].

This patient showed persistent HBV replication as the major determinant of the emerging genomic mutations [7,25].

Entecavir is a 100-300 fold more potent inhibitor of the wild-type viral polymerase compared to lamivudine-resistant polymerase, when the sensitivity is decreased in a 20-150 fold [26,27]. In this clinical setting, entecavir 1 mg once daily has been proven to be effective in the treatment of lamivudine-resistant patients [9,11]. In presence of the rtL180M and rtM204V as well as entecavir-associated mutations, the susceptibility to entecavir decreased dramatically as seen by an increase in inhibitory concentration 50% (IC_50_) values from 280 to over 1500 fold [28].

Persistent viral replication and the error rate of HBV reverse transcriptase (~10^-4 ^base/replication cycle) are major factors in the development of resistance [29].

The viral polymerase characterization in our patient at quasispecies level showed lamivudine-associated mutations rtL180M and rtM204V/I during adefovir, entecavir and entecavir-tenofovir therapies.

During the entecavir monotherapy we observed no viral suppression and tenofovir 300 mg once daily was added to the therapy resulting in a rapid 2 Log_10 _decline in viral load. This level was sustained for seven months until a viral rebound and biochemical breakthrough were observed. In spite of the fact that the A181T adefovir-resistance and the T184L entecavir-resistance mutations were present in the background, they were only minor components of the HBV quasispecies that emerged once tenofovir was added in two consecutive samples separated by a five-month interval. The alanine at position 181 seems to be critical in the development of resistance to nucleos(t)ide analog, since it is located in α-helix adjacent to the nucleotide binding site [30]. Taking into account that the availability of free replication space is necessary for the spread of the mutant virus, the kinetics of emerging drug-resistant mutants is usually slow. Such HBV variants became neither dominant in the two highly replicative (~10^8 ^IU/ml) viral populations nor were detected three months later. The intrinsic resistance of the mutant and its replicative fitness could explain the time to its emergence as well as the ephemeral contribution to HBV quasispecies. In addition, taking into account that the clonal analysis was performed on some samples with low viremia levels, the consequent reduction in the number of viral species detected could hamper the interpretation of results.

This behaviour in HBV is remarkably different from that described in two recently reported cases showing entecavir resistance with a similar therapeutic background [31,32]. The A194T mutation that appears to lead to an over ten-fold decrease in tenofovir sensitivity [33] was not detected in the HBV quasispecies composition of those previous isolates during entecavir-tenofovir therapy.

The combination of the rtL180M+ rtM204V mutations harbored by our patient results in a replicative fitness of about 50% of that shown by the wild-type virus [28,31]. Additional mutations able to restore replicative fitness to similar levels in the wild type such as rtV173L and rtP177S were not found. These factors may explain, in part, the difference in resistance rates between entecavir and lamivudine, as more profound genetic changes are necessary for a mutant to become the dominant strain. The role of the rtA200V and rtI253V mutations found during lamivudine therapy is not clear. Their impact on sensitivity and replication fitness is unknown, but nevertheless, they remained present during entecavir monotherapy or combined entecavir-tenofovir therapy, which may imply a certain influence of these mutations [34]. Future *in vitro *research should clarify their impact on drug sensitivity and replication fitness.

In spite of the fact that adefovir and tenofovir are active against lamivudine resistant mutants, their activities decrease [14,27,35,36], and lamivudine mutations appear to have an impact on the therapeutic efficacy of adefovir in this clinical setting [6,37]. Also, it is important to consider that adefovir monotherapy in lamivudine-experienced patients is a treatment modality that carries a significant risk of resistance in the long term. Current guidelines recommend that it could be overcome by the adefovir-lamivudine combination therapy [38].

Considering that HBV viral load exhibited intermittent long-term fluctuations under a constant therapy during the follow-up, an inadequate adherence appears to be a plausible cause for treatment failure since an inadequate absorption or altered metabolism does not seem to impact in this case in view of the concentration of drug found in plasma [39]. The viral load decline was fast in the short term, which additionally supports a probable adequate drug metabolism but it needs to be determined by further accurate pharmacokinetic studies.

Based on our findings and the available data on HBV resistance, and in *lieu *of the fact that tenofovir is an appropriate therapeutic alternative for entecavir-refractory patients, in this case tenofovir was not efficacious.

## Conclusion

This case shows that the lack of response to tenofovir combined with entecavir can occur in lamivudine-adefovir refractory patients chronically infected with HBV with incomplete viral suppression. On the basis of our findings, patients in whom tenofovir is used as a treatment option for entecavir-resistant hepatitis B should be closely monitored.

## Consent

Written informed consent was obtained from the patient for publication of this case report. A copy of the written consent is available for review by the Editor-in-Chief of this journal.

## Competing interests

The authors declare that they have no competing interests.

## Authors' contributions

LC, SB, FF, HT and JQ: have been involved in acquisition and interpretation of data. HT has been involved in patient clinical care, and LC, JQ have been involved in drafting the manuscript. LC, SB, and FF carried out the standard and specific microbiologic tests and the molecular genetic studies. JQ, HT and SB reviewed the manuscript. All authors read and approved the final manuscript.

## Pre-publication history

The pre-publication history for this paper can be accessed here:

http://www.biomedcentral.com/1471-2334/11/178/prepub
